# The oxygen dissociation curve of blood in COVID-19–An update

**DOI:** 10.3389/fmed.2023.1098547

**Published:** 2023-02-27

**Authors:** Dieter Böning, Wolfgang M. Kuebler, Dominik Vogel, Wilhelm Bloch

**Affiliations:** ^1^Institute of Physiology, Charité Medical University of Berlin, Berlin, Germany; ^2^Klinik für Interdisziplinäre Intensivmedizin, Vivantes Humboldt-Klinikum, Berlin, Germany; ^3^Department of Molecular and Cellular Sport Medicine, Institute of Cardiovascular Research and Sport Medicine, German Sport University Cologne, Cologne, Germany

**Keywords:** hemoglobin oxygen affinity, sickle cells, anemia, erythroblasts, *in vivo* oxygen dissociation curve

## Abstract

An impressive effect of the infection with SARS-Co-19 is the impairment of oxygen uptake due to lung injury. The reduced oxygen diffusion may potentially be counteracted by an increase in oxygen affinity of hemoglobin. However, hypoxia and anemia associated with COVID-19 usually decrease oxygen affinity due to a rise in [2,3-bisphosphoglycerate]. As such, COVID-19 related changes in the oxygen dissociation curve may be critical for oxygen uptake and supply, but are hard to predict. A Pubmed search lists 14 publications on oxygen affinity in COVID-19. While some investigations show no changes, three large studies found an increased affinity that was related to a good prognosis. Exact causes remain unknown. The cause of the associated anemia in COVID-19 is under discussion. Erythrocytes with structural alterations of membrane and cytoskeleton have been observed, and virus binding to Band 3 and also to ACE2 receptors in erythroblasts has been proposed. COVID-19 presentation is moderate in many subjects suffering from sickle cell disease. A possible explanation is that COVID-19 counteracts the unfavorable large right shift of the oxygen dissociation curve in these patients. Under discussion for therapy are mainly affinity-increasing drugs.

## 1. Introduction

Infection with SARS-CoV-19 causes multiple organ failure. An especially impressive effect is the impairment of oxygen uptake. The underlying general pathophysiology of COVID-19 has been previously reviewed by various authors, e.g. (1, 2). The impairment of oxygen uptake specifically relates to (a) impaired oxygen diffusion due to edema formation in the injured lung, (b) V/Q mismatch due to microvascular thrombosis, loss of hypoxic pulmonary vasoconstriction, or bronchopulmonary anastomoses, and (c) impaired oxygen transport in blood due to anemia resulting from cell damage or reduced cell production. In addition, ECMO treatment may cause hemolysis and a rise in COHb concentration. Further, changes in oxygen affinity of hemoglobin occur [reviewed by 3] which may affect oxygen uptake and delivery: In the lung an increase in affinity may facilitate O_2_ uptake while oxygen delivery to consuming cells is enhanced by decreasing affinity.

Pubmed searches for COVID-19 AND hemoglobin (1,090 articles), COVID-19 AND erythrocytes (559 articles) or COVID-19 AND erythrocyte function (310 articles) yield a large body of publications. Most decisive for red cell function is, however, hemoglobin oxygen affinity. Yet, only 14 publications address COVID-19 AND hemoglobin oxygen affinity, which is still 2 times more than in the first quarter of 2021, when we wrote our first review (3). Only 8 of these publications, however, contain actual measurements, the remaining are reviews, comments or letters to the editor. Some aspects in the more recent articles are new and help to refine our understanding of oxygen uptake, transport and delivery in COVID-19. In particular, the presence of the ACE2 receptor in part of the erythroblasts which allows binding of SARS-CoV-2 may be relevant (4). Similarly, binding of SARS-CoV-2 to Band 3 has been suggested by *in silico* data. Before discussing the most recent findings on the oxygen dissociation curve, a description of the physiological basis and a summary of the previous review are useful.

## 2. Determinants of hemoglobin oxygen affinity

Oxygen affinity is an intrinsic property of hemoglobin in red blood cells that can be modified by a variety of factors [reviewed e.g., by 5]. For determination of oxygen affinity the oxygen dissociation curve (ODC) has to be measured. Characteristic properties of the ODC are the half saturation pressure P_50_ and the slope n (see below) in the logarithmic Hill plot. A low P_50_ is favorable for oxygen binding to Hb in the lung capillaries, while a high P_50_ is favorable for oxygen delivery to the consuming cells. *In vivo* variations in pH (higher in the lungs than the consuming tissues), PCO_2_ (lower in the lungs) and temperature (lower in the lungs than in tissues with high energy turnover) as well as additional, partly unknown factors may cause such variations in affinity. The resulting *in vivo* ODC is therefore steeper than the standardized *in vitro* curve (see below).

Various factors are important for oxygen affinity. There are intraerythrocytic (e.g., cell age) and extraerythrocytic (e. g. plasma concentrations of influencing substances) effects. An overview is presented in Table 1.

**TABLE 1:** Internal (erythrocytes) and external (plasma and interstitial fluid) regulators of Hb-O_2_ affinity.

Parameter	Change of P_50_	Known change during COVID-19
**Internal**		
Type of Hb	∧ or ∨	No change
2,3-BPG	∧	Slight increase?
ATP	∧	∧?
GSH	∨	No change
Production of acids (e.g., lactic acid)	∧	Possibly increased
Cellular concentration of non-diffusing substances (Donnan equilibrium)	∧	?
Non-diffusing anions (Donnan equilibrium)	∨	Depending on plasmaprotein concentration
Cell age	∨	?
**External**		
Temperature	∧	∧
Osmolality	?	?
pH	∨	Variable
PCO_2_,	∧	Variable, initially low
HCO_3_^–^	∨	Variable
Other diffusible anions (mainly Cl^–^. La^–^)	∧	Variable
Non-diffusing anions (Donnan equilibrium)	∨	Depending on plasmaprotein concentration

Data from sources evaluated in Böning et al. (3) and Mairbäurl and Weber (5).

### 2.1. Intraerythrocytic effects

Besides the type of Hb (HbA in most subjects), the following substances produced in the red cells are especially relevant: While 2,3- bisphosphoglycerate (2,3-BPG) and adenosin triphosphate (ATP) increase P_50_, glutathione (GSH) reduces it moderately. A new analysis of the GSH effect was published recently (6). Additionally all not freely diffusing anions (especially Hb^–^ because of its high concentration) influence the Donnan equilibrium and increase the intraerythrocytic [H^+^].

### 2.2. Extraerythrocytic effects

In addition to intraerythrocytic factors, properties of blood plasma are also influential. In contrast to *in vitro* investigations exchange with the interstitial space is relevant *in vivo* especially in the oxygen consuming tissues. In COVID-19 the small extracellular space in the lungs may be increased by the accumulation of interstitial or even alveolar edema fluid. The concentrations of CO_2_, Cl^–^, lactic and other fixed acids as well as water (osmolality) in the erythrocyte equilibrate with the surrounding fluid; but because of the Donnan effect especially of Hb the concentrations of freely exchangeable anions are lowered compared to the plasma. All acids and bases influence the pH, but those entering the red cell (e.g., CO_2_, Cl^–^, lactic acid) can additionally bind to Hb at its end-terminal nitrogen. Lactic acid is produced not only in the muscles, but also within the red cells and interchanges slowly after binding to the monocarboxylate-transporter 1 (7–9). A relevant influence of nitric oxide on hemoglobin oxygen affinity on the other hand is disputed because of its very low concentration compared to Hb (5).

## 3. Methods for the determining O_2_ affinity

The classical method for the determination of O_2_ affinity is measurement of oxygen saturation (SO_2_) or content *in vitro* after equilibration of blood with varying PO_2_ at standard conditions (pH 7.4, PCO_2_ 40 mmHg, temperature 37°). While PCO_2_ and temperature can be set by the investigator, pH varies slightly because of the Haldane effect and has to be corrected for (see below). These *in vitro* conditions resemble the situation in the healthy lungs with a very small extracellular volume. The influence of temperature and acids can be studied by equilibration at different conditions (PCO_2_, temperature) or after addition of acids or bases (e.g., HCl, lactic acid). A drawback is the required volume of blood; approximately a minimum of 5 ml is necessary for one curve with five points when using sphere tonometers. We have previously reduced this to 2–3 ml for 10 points by adding oxygen to desaturated blood in a syringe (10). A frequently used apparatus is the Hemox-Analyzer (11–13) where only 50 μl of blood are added to 50 ml buffer solution (100 mmol/l sodium chloride, 5 mmol/l potassium chloride, 30 mmol/l imidazole buffer, 5 mmol/l sodium phosphate, 5 mmol/l glucose) and equilibrated with oxygen or nitrogen. Advantages are the ability to investigate minimal red cell volumes as well as isolated erythrocytes, and possible manipulation of experimental conditions. The lack of CO_2_ in this assay has rightfully been criticized by Harutyunyan et al. (14). In addition, the buffer lacks Ca^++^, bicarbonate, proteins, and lipids. Further, substances in the plasma which interchange with red cells or influence the membrane potential (e.g., bicarbonate and lactate) are largely diluted resulting in their efflux from the erythrocytes into the buffer solution. As such, characteristic *in vivo* effects of these compounds may completely disappear *in vitro*. Some of the drawbacks can be abolished by using a plasma-like solution and adding CO_2_ to the equilibration gas, as previously described by e.g., (15). A new apparatus where only some microliters of undiluted native blood are necessary might be useful in the future (16).

Because of lacking laboratory facilities, small available blood volumes or risk of infection, most investigators of COVID-19 have calculated P_50_ values from single blood samples after routine blood gas analysis. The following equations published by Severinghaus (17) are most often applied for correction to standard conditions (pH 7.4, base excess 0 mequ/l, 37°C):

Correction for Bohr shift by CO_2_ and fixed acid (e.g., lactic acid):


(1) $$\Delta \log{\text{PO}}_{2}=-0.48\Delta \mathrm{pH}+0.0013\Delta \mathrm{base}\mathrm{excess}$$

Correction for temperature shift:


(2) $$\Delta \log{\text{PO}}_{2}=0.024\Delta T$$

Presentation in the Hill diagram (18):


(3) logSO/2(100-SO)2=n*logPO2+K

The slope n is rather constant over a large range of SO_2_ [e.g., (19)] allowing to calculate a standard P_50_ from one pair of PO_2_ and SO_2_ measurements. However, n may be influenced by additional factors such as physical training (20) or illness, e.g., peripheral artery disease (21) or cystic fibrosis (19).

Yet, various modulating factors were initially not known and are therefore not considered in these equations, especially the effect of organic phosphates like 2,3-BPG (22, 23) and the modulation of the Bohr effect as a function of oxygen saturation at values below 20 as well as above 90% SO_2_ (24, 25). To avoid influences of the latter, Severinghaus (17) suggested to use only samples between 20 and 80% SO_2_ for the P_50_ calculation. As long as the deviations from the standard conditions are small, these effects are of no major relevance. A more important problem is, however, whether these factors are equally large *in vivo*. Specifically, we have observed considerably *in vivo* deviations of P_50_ as well as of the Bohr coefficients in various studies [discussed e.g., in (19, 26)], which may be attributable to the constant exchange of substances between blood, interstitial fluids, and even parenchymal cells.

## 4. Summary of our first review

In our initial review on the topic (3), four papers containing measurements of oxygen affinity were considered (27–30). The techniques used in these for the determination of O_2_ affinity were either the Hemox-Analyzer or calculation from single arterial or venous blood samples applying Severinghaus’ equations to obtain the standard half saturation pressure P_50_.

In these reports, no right shift of the ODC was detected as would normally be expected in patients with hypoxia due to a counterregulatory increase in [2,3-BPG] (5). The largest investigation (30) even showed a left shift calculated from thousands of venous or arterial blood samples (23.4 mmHg compared to the accepted value of 26.7 mmHg in healthy subjects). This was astonishing since the patients additionally suffered from marked anemia. Anemia might cause an increase in P_50_ to more than 30 mmHg resulting from high 2,3-BPG concentrations (15); additionally Hill’s n tended to higher values at SO_2_ above 50% in this investigation. At that time, we speculated that a high concentration of methemoglobin (MetHb) as observed in some investigations [summarized by Scholkmann et al. (31)] might have reversed the anemia effect in COVID-19 patients and caused a left shift of the ODC. Additionally we suggested that various commonly observed, yet not very well-understood *in vivo* effects might have caused a further left shift in Vogel’s (30) investigation.

In this context, the following methodological problems have to be considered: P_50_ in COVID-19 patients was compared to values obtained in patients with other (non-COVID) diseases, while a comparison with healthy subjects would have been preferable. Measurement of complete ODCs at different physiological conditions (pH, PCO_2_, temperature) with established methods especially including determination of 2,3-BPG had not been performed. The latter has been measured only in one study on erythrocytic metabolism in moderate COVID-19, but only arbitrary units were presented hinting to slightly elevated concentrations (32). Reasons for the surprising paucity of data on [2,3-BPG] in COVID-19 might be that the appropriate commercial test kits are no longer available and that the physiological laboratories specialized in [2,3-BPG] measurements are not prepared for work with infectious samples.

## 5. New publications on hemoglobin oxygen affinity in COVID-19

Pubmed and Google Scholar were searched up to December, 2022 for “COVID-19 AND oxygen dissociation curve” as well as “COVID-19 AND hemoglobin oxygen affinity.” An overview is presented in Table 2.

**TABLE 2:** Oxygen affinity in COVID-19 patients.

Authors	COVID-19 group	Comparison group	P_50_ (mmHg) COVID-19	P_50_ (mmHg) comparison	Signif. *p*	[Hb] (g/dl)	Method	Annotations
De Martino et al. (27)	12m, 9f	10m, 11f ARDS	Data lacking	26.7	ns	∼13	G	v. No clear deviation from theoretical standard ODCs, but tendency for a right shift
Daniel et al. (28)	7m, 7f	5m, 6f no diagnosis	29.0 ± 2.3	28.5 + 1.8	ns	9.3 ± 2.3 14.3 ± 1.1	H	Anemia in COVID-19 patients
Renoux et al. (29)	7	7 healthy 7 sepsis	29.7 [27.2; 31.0]	26.7 [26.3; 29.3]; 27.6 [27.0; 30.6]	ns	11.6 [9.0; 14.4] 10.7 [9.8; 12.4]	H	v. SO_2_ of all and Hb of healthy subjects not communicated
Vogel et al. (30)	43m and f	828 critically ill	23.4 ± 3.1	24.6 ± 5.4	< 0.001	8.1 ± 1.2 9.4 ± 2.0	C	a and v. Comparison to standard P_50_ 26.7 *p* < 0.0001
Gille et al. (38)	70m, 30f	69m, 31f histor. control	26 [25.2–26.8]	25.9 [24.0–27.3]	ns	14 [12.6; 15.2] 13.2 [11.4; 14.7]		a. Diagnosis in historical control not communicated!
Pascual-Guàrdia et al. (42)	79m, 60f	73, historical control or negative PCR	not calculated	not calculated		13.3 ± 1.8 11.8 ± 2.4	C	a. P_50_ not calculated, but many PO_2_ values differ > 2 SD from mean. Sex of comparison patients not communicated.
Pascual-Guàrdia et al. (42)	110m, 105f	379, historical control or negative PCR	not calculated	not calculated			C	v. P_50_ not calculated, but many PO_2_ values differ more than 2 SD from mean. Sex of comparison patients not communicated.
Laredo et al. (45)	89m, 35f	104m, 37f	27.4 ± 0.3	27.5 ± 0.2	< 0.05	11.1 [9.6; 13.2] 10.8 [9.4; 12.7]	C	Probably a and v. Lower Bohr coefficient in COVID-19.
Ceruti et al. (50)	25 survivors	7 deceased	23.0 ± 1.6	32.2 ± 7.9	< 0.01	11.5 ± 2.0	C	a. Comparison deceased vs. survivors
Hlutkina et al. (51)	15	15 healthy	31.8 [29.5; 34.3]	27.9 [27.5; 28.9]	< 0.05	12.1 [9.2; 13.0] 14.1 [11.7; 14.4]	C	v. SO_2_ not communicated
Valle et al. (56)	517	314	26.3 ± 1.2 vitro 25.7 ± 1.4 vivo	26.8 ± 2.9 vitro 27.1 ± 2.8 vivo	0.001 0.001	13.0 ± 1.8 13.0 ± 2.3	C	a. Higher survival in patients with initial P_50_ < 27 mmHg
Bergamaschi et al. (57)	289m *In vivo*	289m *In vitro*	25.4 [24.5–6.4] *in vivo*	27.2 [26.3–28.2] *in vitro*	0.01	12.9 [11.7–141]	C	Mild form of illness, age 75 (63–82) years

Explanations: m, male; f, female; ARDS, viral pneumonia-induced acute respiratory distress syndrome; ns, not significant. P_50_ calculated for standard conditions. vitro, in vitro; vivo, in vivo. Methods: H, Hemoxanalyzer; C, calculation from arterial or venous measurements by applying Hill or Severinghaus equations; D, calculation from arterial or venous measurements by applying Dash equations; G, graphical representation in the SO_2_-PO_2_ diagram. Annotations: a, arterial; v, venous.

Early after the publication of our review Vogel et al. (33) communicated that they had meanwhile measured methemoglobin levels and detected no increase in the majority of cases. Specifically, MetHb exceeded 3% in only 30 of 3,518 samples. This finding was also supported by Gille et al. (34) in a comment (see below). In other studies, however, elevated MetHb levels might play a relevant role: Alamdari et al. (35) reported an average of 16.4 ± 9.1 SD% in 21 severely ill patients treated in the intensive care unit. Caution is warranted as some MetHb formation may also occur during the time between blood sampling and measurement. In our own investigations (30) measurements were performed immediately after blood sampling. Alamdari et al. (35), however, state that their samples were transported on ice to the laboratory. But formation of MetHb is an autoxidative process without molecule collisions which cannot be prevented by cooling. As such, MetHb may cause a left shift of the ODC in patients with COVID-19, especially when treated with drugs favoring MetHb formation such as chloroquine and hydrochloroquine. These substances were not applied in the patients studied by Vogel et al. (30).

Hence, the cause for the low P_50_ in Vogel’s paper remains unknown and the measurement of 2,3-BPG becomes ever more important. Unfortunately, we had to recognize that previously utilized commercial test kits are no longer produced (36), which is likely the reason why none of the above mentioned papers on oxygen affinity in COVID-19 with the notable exception of the report by Thomas et al. (32) measured [2,3-BPG], and even the Thomas paper determined only relative concentrations (Personal communication A. D’Alessandro). As such, it remains unclear whether the detected left shift in Vogel′s study occurred because of a lack of the (physiologically expected) increase in 2,3-BPG or whether it was caused by unknown factors despite elevated [2,3-BPG]. Various substances (e.g., ATP, Cl^–^, lactate, glutathione) may have influenced oxygen affinity in this investigation (36). The concentration of NO in blood is low but its binding mechanism to Hb is similar to MetHb formation at high oxygen saturation; as such NO may possibly influence the position of the ODC during the lung passage (37); this effect, however, disappears at low oxygen saturation in the tissue capillaries.

While our first review was in press, Gille et al. (38) published a similar study as Vogel et al., but did not detect a change in the ODC in their COVID-19 patients compared to a non-uniform group of patients with infection, airway disease, interstitial lung disease, or heart failure. MetHb was also not increased, similar to Vogel’s investigation. Subsequently the authors also wrote a letter to the editor with respect to our review (34). A careful analysis of both papers yields the following conclusions (39):

(1) Gille et al. evaluated data from 100 patients with COVID-19 and compared them to 100 patients with acute respiratory failure (infection, airway disease, interstitial lung disease, or heart failure) who had been assessed prior to the emergence of the COVID-19 pandemic. They did not detect differences in the P_50_ [median 26 (25.2–26.8) versus 25.9 (24–27.3) mmHg]. The number of measurements was, however, much smaller than in Vogel‘s study (19.463 from 43 COVID-19 patients and 828 critically ill patients with acute respiratory failure), and the average Hb concentration was markedly higher (median 14.0 g/l versus mean value 8.1 g/dl).

As PO_2_ scatters markedly at saturations higher than 97%, Gille et al. excluded these values. According to various investigations, [e.g., (24, 25)] also the Bohr effect is markedly reduced above 90% SO2. Therefore, this exclusion is reasonable.

Vogel et al. (30) did not exclude data, instead, they evaluated all measurements between 20 and 100% SO_2_. Mean values of approximately 94% SO_2_ were similar for COVID-19 patients and the control group. The Bohr effect correction was negligible (pH 7.382 ± 0.077 SD). Outliers were present in both groups, therefore the detected P_50_ difference can be assumed to be real. One may conclude that the different results in the studies by Vogel et al. and Gille et al. do not seem to be caused by different methods, but to depend on other causes such as different severity of the illness; this notion is supported by the fact that all patients in Vogel’s study received ventilator support, and that [Hb] was much lower than in Gille’s study.

In Figure 2 Gille et al. present the time course of the standard P_50_ over 18 days in COVID-19 patients and non-COVID patients. They do not find a significant difference, albeit a tendency for lower mean values in the COVID-19 patients relative to the control group is visible (e.g., a 2 mmHg difference on days 8–10). Possibly the number of measurements (maximally 15 on each day) was too low or the variability of diseases in the non-COVID patients (infections, airway disease, interstitial lung disease, heart failure, surgical interventions) too high. In any case lack of significance does not proof the absence of an effect.

Surprisingly Gille et al. added COHb to O_2_Hb for the calculation of SO_2_. This is not correct in our eyes, but used in some apparatus (40). Accordingly, the calculated P_50_ is lowered, but [CO-Hb] is rather small in this investigation and therefore the effect presumably negligible.

Additionally, 55 subjects with high CO-Hb and 30 subjects with sickle cell disease (see below) were studied. As expected, the ODC was left shifted in the CO-Hb group.

(2) Similar to the previous report by Vogel et al., the findings of Gille et al. exclude MetHb as main factor for a left shift of the *in vivo* ODC in COVID-19. As mentioned above, a possible influence of variable NO-binding to Hb might yield an alternative explanation for the reported left shift (36). On the other hand, erythroblasts express ACE2 on the plasma membrane and are therefore potentially vulnerable to SARS-CoV-2 infection in cases of viremia (41); this, again, might influence oxygen affinity.

Pascual-Guàrdia et al. (42) published a rather large investigation (approximately 1,100 arterial or venous samples) in COVID-19 patients who in most cases were not critically ill (no mechanical ventilation, mean Hb concentrations of 13.3 ± 1.8 SD g/dl) and compared them to an even larger group of patients with pulmonary dysfunction caused by other illnesses. The authors did not observe a significant change in oxygen affinity, but scattering was very large. In 35 venous samples the difference between measured and calculated SO_2_ (by use of three published standard curves) was larger than two standard deviations (approx. ± 6%).

In a letter to the editor we (43) therefore suggested that changes in Hb-concentration or other indicators of severe illness (e. g. mechanical ventilation) might have influenced P_50_ in these patients. Possibly the effects of critical illness with profound anemia were similar to those reported by Vogel et al. (30), and might thus have caused the large scatter in the study by Pascual-Guàrdia et al.

Subsequently Pascual-Guàrdia et al. (44) presented data for severely ill patients only. In this group, again, no significant change in P_50_ was detectable, but the scattering remained improbably large. Therefore, the data of Pascual-Guàrdia neither supports nor contradicts those measured by Vogel et al. (30).

Laredo et al. (45) published a letter to the editor reporting a large investigation comparing patients treated in the intensive care unit (5,291 measurements in COVID-19 patients in 2020 versus 3,449 measurements in Non-COVID patients in 2018–2019); more than 60% were mechanically ventilated. The reference group suffered from bacterial pneumonia (*n* = 80, 56%), influenza (*n* = 32%), and non-infectious injuries; this selection is similar to that by Gille et al. (38). Average arterial SO_2_ was high in both groups [95.7 (93.9–96.8)% in COVID-19 patients vs. 95.1 (91.7–96.3)% in Non-COVID patients with slightly higher values in COVID-19 patients (*p* = 0.03), the corresponding PO_2_ amounted to 107 mmHg (91–128) and 95 mmHg (77–115), respectively (*p* = 0.002)]. Yet, at least 100 SO_2_ values were lower than 70%. The medians of PCO_2_ [43 mmHg (38–48) vs. 42 mmHg (37–49), (*p* = 0.87)] and pH [7.41 (7.38–7.45) vs. 7.40 (7.34–7.44), (*p* = 0.04)] were in the normal range, but individual values scattered considerably.

The authors applied a relatively complicated equation for P_50_ calculation: the constants for correction to standard conditions (P_*CO*2_ = 38 mmHg, pH = 7.4) were obtained from each patient′s data, for comparison the median was used. Hb concentration amounted to 11.1 [9.6–13.2] and 10.8 [9.4–12.7] g/dl, respectively, corresponding to moderate anemia.

In COVID-patients, the calculated standard curves were only slightly yet significantly left-shifted by 0.1 mmHg as compared to the reference group (27.5 ± 0.2 SD mmHg). This shift is physiologically negligible. Interestingly, however, the calculated Bohr coefficient for CO_2_ was slightly reduced in COVID-19 patients, which reduces the *in vivo* P_50_ further.

The main problem with P_50_ calculation in this investigation is that many PO_2_ were very high (up to > 300 mmHg in Figure 1 of Laredo’s article). The large scattering as well as the disappearance of the Bohr effect reduce the probability of a correct P_50_-calculation from these values. Laredo er al. did not exclude values above 95% like some other authors. But when considering all single standardized values (SO_2_ versus PO_2_) presented in Figure 1 of Laredo’s article, it is visible that below 95% saturation the majority of points in the COVID-patients are left-shifted compared to the Non-COVID group.

To summarize: This investigation shows only a minor effect of COVID-19 on the ODC, but experimental limitations may have obscured a potentially larger effect. Specifically, a potential left shift at high oxygen saturation and a corresponding right shift at low saturation levels may have cancelled each other out and resulted in a seemingly unchanged mean value.

An article possibly clarifying the discrepancies in preceding publications might be “Temporal Changes in the Oxyhemoglobin Dissociation Curve of Critically Ill COVID-19 Patients” by Ceruti et al. (46). Arterial blood gases were repeatedly assessed in the intensive care unit (3,514 analyses in 32 patients) and the standard P_50_ values of the first 3 days were compared to those measured over the last 3 days. Most patients showed a left shift similar to the study by Vogel et al. (30). A difference between early P_50_ and late P_50_ was detected (20.63 ± 2.1 vs. 18.68 ± 3.3 mmHg, *p* = 0.03 for all patients); however, when values of deceased patients (the number is not communicated) were analyzed, an increase in median P_50_ was observed compared to data in survivors (24.1 vs. 18.45 mmHg, *p* = 0.01).

This decrease of arterial P_50_ in the majority of patients which supports oxygenation in the lungs might be an important advantage for survivors and explain the differing results in former studies. Unfortunately, however, these P_50_ values are improbably low which we have criticized in a letter to the editor (47). Ceruti et al. (46) used equations published by Dash et al. (48) which are rather complicated compared to those of Severinghaus. As they presented no comparing measurements in healthy subjects, a check for a possible calculating error was impossible. Yet, we had never seen such low values in human blood of adults with normal HbA. According to Duhm (49) the standard P_50_ for human red cells completely depleted of 2,3-BPG decreases to only 16 mmHg.

We have therefore recalculated the results using the Hill equation (equ. 3) after correction to standard conditions and assuming *n* = 2.7 (47). When applying this procedure to Ceruti’s data for all patients in their Table 1 (probably means of initial values), we obtained an average P_50_ of 26.9 + 1.9 SD mmHg, which is almost equal to Severinghaus’ standard value of 26.7 mmHg. Similarly, the individual values for each patient in their Table 2 were much higher after recalculation (22.9–32.3 mmHg) than those given in Ceruti’s paper. Only 7 single values were lower than 25 mmHg. We evaluated also the data for the 25 and the 75% percentile of SO_2_ in Ceruti’s Table S1. The P_50_ calculated from these values are lower (23.0 and 22.5 mmHg) but still higher than the means in Ceruti’s paper (initially 20.6, at the end 18.7 mmHg). Exclusion of samples with SO_2_ higher than 95% to avoid the large scattering of PO_2_ did not change the mean standard P_50_.

As such, it became obvious that the P_50_ values calculated by Ceruti et al. cannot be correct. Yet, the observed temporal decrease in survivors is likely real as a systematic error should similarly affect all calculated values during the stay on the intensive care unit. Unfortunately, a control group of patients with other illnesses as in the paper by Vogel et al. (30) was not included in this study.

In their answer to our letter, Ceruti et al. (50) conceded that the originally reported low P_50_ values resulted from a calculation error and announced a revised paper. They communicated that at the end of the stay surviving patients presented a P_50_ of 23.0 + 1.6 compared to 32.2 + 7.9 mmHg in deceased patients. A possible cause might be that anemia was less severe in the recovered patients and therefore [2,3-BPG] concentration lower.

One recently published paper by Hlutkina et al. (51) shows an increase in the standard P_50_ in 15 patients at admission to the hospital compared to 15 healthy subjects (median 31.8 versus 27.9 mmHg) calculated from venous measurements with rather low saturations (median 60%). The authors suggest an effect of increased NO concentrations, which had been previously reported by Mortaz et al. (52).

Since right shifts of the ODC in venous blood have been observed during exercise, e.g., (20, 53, 54) as well as in disease (21, 55), this is not entirely surprising. Such a right shift facilitates oxygen delivery to consuming tissues, yet it simultaneously impacts oxygen uptake in the lungs. Notably, in the investigation by Vogel et al. (30) the reported left shift is only visible in samples with saturations above 50%. Yet, when we recalculated P_50_-values from the medians in the article of Hlutkina and Zinchuk applying Severinghaus’ equations for correction to standard values and Hill‘s equation for calculation of P_50_, we obtained for both groups 30.1 mmHg. We contacted the authors who assured us that their measurements and calculations were correct. A possible explanation for this discrepancy might be that the medians for PO_2_, SO_2_ and pH were measured in different blood samples, a problem which does not exist for mean values. Yet, as a consequence the results of this article have to be taken with a huge grain of salt. In Vogel′s et al. article (30) the left shift is not very pronounced when calculated from means, but clearly visible in the apparently not normal distribution of the single values.

The last paper that came to our knowledge was written by Valle et al. (56). They evaluated arterial blood gas measurements below 92% SO_2_ for P_50_ calculation at entry to the hospital before treatment in 552 COVID-19 patients (75 were accepted into the intensive care unit) and 314 non-COVID respiratory patients applying the equations also used by Vogel et al. (30). Mean standard P_50_ was slightly lower in COVID-19 patients (26.3 ± 1.2 versus 26.8 ± 2.9 mmHg, *p* < 0.001). This difference was more pronounced for *in vivo* conditions, i.e., blood pH, temperature, PCO_2_, and carboxyhemoglobin levels of the patient (25.7 ± 1.4 versus 27.1 ± 2.8 mmHg, *p* < 0.001). In spite of this still small general effect arterial oxygen content was markedly higher in COVID-patients compared to the Non-COVID group with *in vivo* P_50_ below 27 mmHg [17.2 (15.7–18.7) vs. 15.2 (13.0–16.6) mL/dL (*P* < 0.001)]. Interestingly, however, [Hb] was negatively correlated with both *in vitro* and *in vivo* P_50_ showing that the known right-shift effect of anemia was preserved. The prevalence of hypoxemia (73.3 vs. 57.3%, *p* < 0.001), hypocapnia (59.4 vs. 35.7%, *p* < 0.001), combined hypoxemia and hypocapnia (59.4 vs. 27.8%, *p* < 0.001) and alkalosis (53.4 vs. 30.9%) was greater in COVID-19 patients than in the reference group.

Surprisingly, both P_50*s*_ and P_50*i*_ increased significantly over 18 days in the hospital in a subgroup of 33 subjects with regular measurements every 3 days from 25.8 to 27.5 mmHg. The percentage of patients with P_50*i*_ < 27 mmHg decreased from 78.8% at baseline to a minimum of 21.2% 6 days later and still amounted to only 45.5% after 18 days. In 6 patients P_50_ rose by more than 4 mmHg. An important result is that mortality was significantly lower (12.9 versus 23.7%, *p* = 0.014) in patients with low (< 27 mmHg) as compared to those with high P_50*i*_ (> 27 mmHg) at entrance to the hospital.

It seems astonishing that these relatively small changes may have affected survival, yet it should be considered that in the lungs at approximately 90% saturation the PO_2_ differences are increased (see Table 3). It is also possible that the changes are larger during movements or vary during the day. Since anemia was positively correlated with P_50_, this seems to be an additional factor for bad outcome.

**TABLE 3:** PO_2_ to obtain 90% oxygen saturation in dependence on P_50_ (all values in mmHg).

P_50_	PO_2_ for 90% SO_2_	Difference against P_50_ = 27 mmHg
24	54.2	−6.8
25	56.4	−4.5
26	58.7	−2.3
27	60.9	0.0
28	63.2	2.3
29	65.4	4.5
30	67.7	6.8
31	69.9	9.0
32	72.2	11.3

Interestingly mortality correlates with body temperature during the stay in the hospital (57). One causal factor might be the decreasing oxygen affinity hindering O_2_ uptake in the lungs when the temperature rises. The authors suggest cooling (e. g. of the inspired gas) as a possible therapeutic means.

Very recently Bergamaschi et al. (58) published a letter to the editor about measurements in 289 COVID-19 patients. They observed no change of *in vitro* standard P_50_, but low *in vivo* P_50_. This effect was more marked in surviving than in deceased patients [25.2 (24.4–26.3) versus 25.8 (24.7–26.9) mmHg; *p* < 0.012].

### 5.1. Comparison of methods

So far, the results from the various studies on hemoglobin oxygen affinity in COVID-19 are rather heterogeneous. A probable cause is that according to Ceruti et al. (46) changes partly depend on the severity or prognosis of the illness. In line with this notion, in the study by Pascual-Guardia et al. (44) the P_50_-outliers mainly belonged to the critically ill group. Further likely causes for these variations may comprise: mixtures of venous and arterial samples, inequality of comparison groups, time delay until measurement, treatment of samples (on ice?), treatment of patients, or acidotic pH in arterial blood due to hypercapnia in severe cases.

Existing studies so far comprise 3 types of analyses: measurement of complete ODCs, calculation of P_50_ from single values determined in arterial blood, calculation of P_50_ from single values determined in venous blood. An overview is presented in Table 2.

### 5.2. Complete ODCs

We detected only 2 publications which measured complete ODCs, both using the Hemox Analyzer. In the study by Daniel et al. (28) P_50_ was rather high (29,0 ± 2.3 mmHg) in 14 COVID-19 patients, but not significantly different from a reference group of 11 patients with unknown diagnosis (28.5 ± 1.8 mmHg). As mentioned above, in this assay all potential *in vivo* effects from soluble factors outside the red blood cells are extremely diluted (1:1000) in the buffer solution. However, [2,3-BPG] in the red cells should remain rather stable during the short-lasting experiments. At first glance one may hypothesize that its concentration should be increased due to anemia (Hb 9.4 g/l). The number of COVID-19 patients is rather small (14) and the reference group consists of severely ill patients, too, yet without anemia (14.3 ± 1.1 g/dl). Renoux (29) measured similar P_50_ values in seven COVID-19 patients, but the values of their reference group consisting of healthy subjects did again not differ. Additional problems might be that variations of buffer composition (and thus different pH) and type of anticoagulant (e. g. acid citrate dextrose) can influence the P_50_ values (59). The standard deviation of the method is ± 1.1 mmHg with the original buffer according to Mawjood (60). General problems for all measurements are storage duration (when did the blood arrive in the laboratory?) and conditions (e.g., storage temperature).

### 5.3. P_50_ calculation from single arterial samples

Three large studies using P_50_ calculation from arterial samples show a left shift of the ODC (30, 33, 46, 56). In two the outcome is positively correlated to a low half saturation pressure (46, 56), in one P_50_ was higher in anemic subjects with a bad prognosis (56). Patients in whom oxygen affinity-and thus, oxygen loading in the lungs - increased over the hospital stay had a higher probability of survival (56). In the patients reported by Ceruti et al. (50) the P_50_ difference between surviving and deceased patients is dramatic: P_50_ of 23.0 + 1.6 compared to 32.2 + 7.9 mmHg in the corrected version of their paper!

### 5.4. P_50_ calculation from single venous samples

Hlutkina and Zinchuk (51) calculated an increased P_50_ in COVID-19 patients from venous blood samples [SO_2_ 59.9 (44.7; 72.8)]. Other authors like Vogel et al. measured also venous saturations but did not present them separately. On their figures, however, a rightward deviation at low SO_2_ from the standard curve is visible. Similarly in the article by DeMartino and colleagues (27) a tendency for a right shift in both patients and control subjects can be seen.

### 5.5. Confounding factors

Further complicating the interpretation of the published results is the fact that the investigated patients are often old and suffering from various additional health problems. On the other hand, the comparison with critically ill subjects with various non-COVID-19 diagnosis may be misleading, if their illnesses also affect hemoglobin oxygen affinity. In various articles the diagnoses and comorbidities of the reference groups are variable or not clearly defined. In chronic obstructive pulmonary disease P_50_ is often markedly decreased (61) due to a systemic inflammatory response.

The changes of the mostly calculated *in vitro* P_50_ for standard conditions are partly rather small and therefore not always statistically provable; *in vivo* the left shift is probably more relevant and, importantly, seems to increase survival rate, as indicated e.g., in 56. When hyperventilation diminishes arterial PCO_2_ leading to a lower *in vivo* P_50_ as is often the case during the initial phase of the illness, this may have a transitory positive result.

Especially problematic is the impaired oxygen uptake in the injured lungs, i.e., in a situation where oxygen saturation is physiologically high. Here, a change of only 1 mmHg in P_50_ reduces or increases the P_*O*2_ necessary to obtain 90% saturation by 2.3 mmHg (Table 3), for 80% saturation the corresponding value is 1.7 mmHg. The same calculation for 95% increases this effect to 3 mmHg. When considering the results of Ceruti et al. (P_50_ of 23.0 ± 1.6 compared to 32.2 ± 7.9 mmHg in surviving vs. deceased patients), the corresponding P_90_ varies by 20 mmHg, a physiologically important effect in injured lungs (50).

### 5.6. Causes for the left shift

Causes for the reported left shift might be reduction of 2,3-BPG and/or concentration changes of other ODC-modulating molecules in the erythrocyte (e.g., ATP, glutathione, chloride, CO_2_). Changes in red cell age because of hemolysis or altered erythropoiesis may also be important: The half saturation pressure of old erythrocytes is reduced due to lower [2,3-BPG] (62). Another factor might be that effective hyperventilation with reduction of alveolar PCO_2_ and an increase in red cell pH as commonly seen in anemia is not possible in many COVID-19 patients. This failure of an adaptive ventilatory response might reduce 2,3-BPG synthesis and thus, promote a left shift in spite of anemia. In any case, even a “normal” standard P_50_ in anemic patients is in fact already a reduced one. Damages of the red cell membrane, as described e.g., in Thomas et al. (32), might change substance concentrations in the erythrocyte or allow exit of Hb molecules which influences affinity (e.g., plasma pH is about 0.2 units higher than cell pH). If erythropoiesis is suppressed because of cell damages already in the bone marrow (see Chapter 6), the proportion of old erythrocytes with low [2,3-BPG] rises resulting in lowered P_50_. Finally MetHb as cause for P_50_ changes probably plays no important role. In 4 of the articles described in this chapter (38, 46, 56, 58) mean values did not exceed 1%.

An interesting aspect is that measurements in venous blood show no significant effect on P_50_ (27, 29) or even a rise in P_50_ (29, 51). This finding is reminiscent of the results of Vogel et al. (30), where the P_50_ values calculated for low SO_2_ tended to lie to the right of the standard curve. Similarly, measurements in venous blood during physical exercise often show a deviation to the right [e.g., (21, 63, 64)]. The causes for this right-shift are not fully explained. A possible cause are shifts of anions (Cl^–^ or HCO_3_^–^), which enter red cells in the peripheral tissues and exit them again in the lungs. The decrease in Cl^–^ might be further enhanced in COVID-19 due to an increase in the pulmonary distribution space resulting from interstitial or alveolar edema fluid.

Astonishingly no author has considered a possibly varied Donnan effect caused by changes in the intraeythrocytic Hb concentration. A reduction of [Hb]_*ery*_ increases pH_*ery*_ resulting in lowered P_50_ when applying pH_*plasma*_ for the calculation. But this effect is probably negligible. In the studies with P_50_ determinations only 3 (29, 30, 46) communicate Hct values necessary for the calculation. The resulting [Hb_*ery*_] is only slightly decreased (mean values 29.7–32.9 g/l compared the normal value of 33 g/l).

### 5.7. Conclusion from chapter 5

According to 4 studies in more than 600 patients COVID-19 has a remarkable effect on the ODC, causing in arterial blood a left, and in venous blood a right shift. This notion is based on measurements in several thousand blood samples. Lacking effects in other (mostly smaller) studies might result from a variety of confounding influences, e.g., comorbidities. Since extended laboratory measurements (complete ODCs and 2,3-BPG determinations) or comparative measurements between laboratories have been rarely performed, exact causes remain unknown.

## 6. Effects of COVID-19 on erythropoiesis and oxygen supply

SARS-CoV-2 infectivity has been demonstrated in erythroid progenitor cells (41). This invasion of erythroid precursors and progenitors by SARS-CoV-2 is a cardinal feature of COVID-19 disease which may in part explain the evolving hypoxia (65). The infection of erythroid progenitor cells can lead to hematopoietic stress which may result in RBC morphological abnormalities, inability to respond to environmental cues, and premature egress from the bone marrow. These findings provide a mechanistic concept for the association of COVID-19 disease with RBC abnormalities. For example, dysregulated iron homeostasis has been reported and unusual RBC morphological abnormalities have been observed in COVID-19 patients. The recognition of RBC precursors as a direct target of SARS-CoV-2 has led to the hypothesis that SARS-CoV-2 induced dysregulation of hemoglobin- and iron-metabolism may contribute to severe systemic courses of COVID-19 (66). The premature egress from the bone marrow may in part reflect a physiological response to hypoxia. Indeed, COVID-19 patients appear to have elevated RBC distribution width (RDW) and altered erythrocyte shape (67). Moreover a differential impact of SARS-CoV-2 variants on erythropoiesis in COVID-19 patients with a more prominent impact of the original Wuhan variant compared with the Delta and Omicron variants has been proposed (68). Altered RBC morphology and composition due to impaired erythropoiesis could be one reason for the modulation of RBC function including oxygen binding in COVID-19. Additional mechanisms by which SARS-CoV-2 infection may affect mature RBCs are presently under discussion and may potentially explain functional alterations including changes in the ODC.

## 7. Possible pathomechanisms underlying functional and structural damage of red blood cells

The viral infection with SARS-CoV-2 causes significant damage to RBCs that are altered in number, size, rigidity, morphology, hemoglobin content, and distribution width. These changes are associated with functional alterations of RBCs such as changes in RBC metabolism, hemolysis, oxidative stress, NO-metabolism, and oxygen dissociation (3, 69). Damages of RBC membranes and cytoskeleton could be involved in several of the functional and structural RBC abnormalities induced by SARS-CoV-2 infection (69). One cause for the morphological alteration of RBCs could be related to the SARS-CoV-2 virus binding to membrane cluster of differentiation 147 (CD147) receptors and Band3 protein, the most abundant transmembrane protein in the RBCs, on the RBC membrane (70, 71). It should be pointed out that experimental proof for the predicted interaction between red blood cells and SARS-CoV-2 based on *in silico* modeling is lacking so far. However, should SARS-CoV-2 indeed bind directly to red blood cells, devastating consequences in terms of hemolytic activity and RBC properties may be expected.

These proposed mechanisms reduce the functional capacity of erythrocytes for oxygen transport and result in the development of tissue hypoxia (70). Oxygen delivery to tissues can also be decreased by hyperviscosity due to impaired RBC deformability and increased oxidative stress in RBCs (67, 72). The mechanisms leading to increased oxidative stress and impaired deformability of RBCs are not fully resolved up to now. Thomas et al. (32) describe several possible mechanisms which could lead to functional and structural alterations of RBCs. The authors propose that increases in glycolytic metabolites in COVID-19 RBCs are consistent with a theoretically improved capacity of hemoglobin to off-load oxygen as a function of allosteric modulation by high-energy phosphate compounds, possibly as an adaptive response to counteract COVID-19-induced hypoxia. The N-terminus of AE1/Band 3 stabilizes deoxyhemoglobin and fine-tunes oxygen off-loading. RBCs from COVID-19 patients may be incapable of responding to environmental variations in hemoglobin oxygen saturation when traveling from the lungs to peripheral capillaries and, as such, may have a compromised capacity to transport and deliver oxygen. Moreover a damage of the N-terminus of AE1 may compromise the RBC′s capacity to inhibit glycolysis and activate the pentose phosphate pathway in response to oxidative stress, making the RBCs from COVID-19 patients more susceptible to increased oxidative stress. ROS can then react with membrane lipids and proteins, causing lipid peroxidation and modifying membrane proteins, resulting in phosphatidylserine exposure on the RBC surface. This membrane rearrangement is expected to generate an imbalance in cation homeostasis and a concomitant decrease in deformability and–together with oxidative damage of the endothelium–provides a mechanistic explanation for the high incidence of thromboembolic complications and coagulopathies in COVID-19 patients (69). Furthermore, RBCs of COVID-19 patients contain increased levels of glycolytic intermediates, accompanied by oxidation and fragmentation of ankyrin, β-spectrin, and the N-terminal cytosolic domain of Band 3 (AE1). Significant alterations in RBC glycolysis in COVID-19 (67) must also be considered in relation to the ODC shift discussed above. Significantly altered RBC metabolism of lipids, in particular short- and medium-chain saturated fatty acids, acyl-carnitines, and sphingolipids, has been observed (73). A further indicator for RBC membrane alterations are changes in the polyunsaturated fatty acid composition of the RBC membrane which correlate with inflammatory marker expression in COVID-19 patients (74). Further, RBCs of COVID-19 patients reveal increased levels of intraerythrocytic NO and reduced amounts of NO in the serum. This finding could be related to the development of silent hypoxia in some cases of severe disease, as the high levels of intraerythrocytic NO may counteract the release of oxygen at the tissue level and provide an explanation for the reported left shift of the ODC. Damage to AE1 and alterations of the erythrocyte membrane and cytoskeleton by SARS-CoV-2 infection are irreversible. As RBCs circulate for up to 120 days without *de novo* protein synthesis capacity, these effects may not only explain alterations of gas exchange and oxygen affinity properties in COVID-19 patients, but also some of the long-lasting sequelae of COVID-19 (32). It can further be assumed that damage of the erythrocyte membrane reduces the life time of red blood cells, and the resulting anemia will aggravate tissue hypoxia in COVID-19 patients.

## 8. COVID-19 and sickle cell anemia

COVID-19 is surprisingly important in patients suffering from Sickle Cell disease (SCD). SCD results from the exchange of one amino acid (valin for glutamic acid) in one or two β-chains of Hb (75). This exchange causes aggregation of Hb molecules impairing oxygen binding and reducing the deformability of the erythrocytes, the latter resulting in microvascular occlusion and hemolysis, e.g., after sequestration in the spleen. End-organ ischemia or even infarction, anemia, and sterile inflammation are subsequent complications. Yet, *Plasmodium falciparum*–the mosquito-borne parasite invading erythrocytes and causing malaria–has minimized ability to complete its reproductive cycle and cause severe disease in heterozygote carriers of the sickle cell trait. This evolutionary benefit has led to the wide distribution of SCD in equatorial Africa and regions with populations originating from slave trade (76).

Sickle cell disease (SCD) patients typically present a large right shift of the ODC, which impedes oxygen loading in the lungs. This property of HbS is further amplified by a high 2,3-BPG concentration (76, 77). Standard P_50_ of up to 42 mmHg have been observed [reviewed e.g., by Milner et al. 1974 (78)], of which 4 mmHg may be caused by 2,3-BPG according to Henry et al. (75). Interestingly, high affinity hemoglobins (HbF and thalassemia Hb) are occasionally also present in the blood of SCD patients and may reduce symptoms (76).

In their recent study on oxygen affinity in COVID-19, Gille et al. (38) also assessed the ODC in 30 subjects with SCD. As expected, the ODC was right-shifted but the extent of this shift was rather low [30 (26.9–31.9) mmHg]. Hydroxycarbamide reduced the right shift [28.2 (27–31.2) mmHg, *p* = 0.014], while blood transfusion had no significant effect. Unfortunately, no information about the state of the transfused blood (2,3-BPG content normal or reduced due to potential loss during conservation) or the time of measurement after transfusion–which may both influence [2,3-BPG]–are provided.

Various authors [e. g. Parsons et al. (79)] consider SCD a complication of COVID-19 based on the common finding of microthrombosis with occlusion of individual capillaries in critically ill patients (vaso-occlusive crisis). Yet, if COVID-19 decreases P_50_ in arterial blood as often observed (see Chapters 4 and 5), this effect should reverse the characteristic right shift of the ODC in SCD. Indeed COVID-19 presentation is mild in children and moderate in many adults suffering from SCD [reviewed by 80]. Measurements of oxygen affinity in these patients are necessary to test this hypothesis.

## 9. Drugs modulating oxygen affinity in COVID-19

### 9.1. Potential therapeutic agents to modify Hb-O_2_-affinity

When exploring potential agents or methods to modify Hb-O_2_-affinity in a therapeutic way, three questions emerge: Firstly, in which patients could it be desirable to modify Hb-O_2_-affinity? Secondly, what should be the targeted P_50_? Thirdly, by which means is it possible to achieve this target without detrimental side effects?

### 9.2. In which patients could it be desirable to modify O_2_-Hb affinity?

To a certain degree impairment of pulmonary oxygen uptake can be compensated by increased work of breathing and in a clinical setting by delivery of oxygen. However, in some patients these compensatory mechanisms reach their limit. Either because work of breathing becomes harmful (81), or because enriching the alveolar oxygen concentration becomes insufficient to outweigh the degree of lung pathology. In these circumstances sedoanalgesia and mechanical ventilation are indicated to reduce the work of breathing (thereby also reducing oxygen consumption) and to deliver oxygen with positive pressure and lung protective ventilation (81, 82). Once this becomes insufficient, too, and other strategies [e.g., proning (83), pulmonary vasodilators] prove insufficient, extracorporeal membrane-oxygenation (ECMO) can be initiated to remove deoxygenated blood from the venous system, enrich it with oxygen outside the human body, and return it to the patient’s circulation (84). However, this is not without risks and its availability is limited especially in a pandemic setting. Hence, it is a subgroup of critically ill patients with severe respiratory failure in whom modification of Hb-O_2_-affinity may be worth exploring.

### 9.3. What should be the targeted P_50_?

Which Hb-O_2_-affinity is optimal for tissue oxygen delivery under various environmental and pathophysiological conditions has been debated for decades (85). In patients with normal oxygen content but impaired cardiac output, decreasing Hb-O_2_-affinity may appear desirable to improve oxygen delivery in peripheral tissue, e.g., during cardiac surgery performed in a state of deep hypothermia (86–88). However, in a hypoxic environment or when pulmonary oxygen uptake is the limiting factor in the oxygen cascade, increasing Hb-O_2_-affinity may be considered the desired target (85).

In his study on physiology at extreme altitudes on Mount Everest carried out in 1981, John B. West described the progressive left shift of the ODC at increasing altitude reaching an *in vivo* P_50_ of 20 mmHg in an individual climber (89). Dominelli et al. studied humans with high affinity hemoglobin and compensatory polycythemia and were able to show that a left shifted ODC mitigated the decline in exercise performance in acute hypoxia through a higher arterial oxygen content (90). The authors concluded that increased Hb-O_2_-affinity is a superior strategy for preserving exercise tolerance in acute hypoxia. Similar conclusions had already been drawn in the 1970s by Eaton et al. (91) as well as Hebbel et al. (92), and more recently by Yalcin et al. (93). According to mathematical modeling, an increase in Hb-O_2_-affinity resulting from a P_50_ change of -3 mmHg only slightly increases SO_2_ (by 1%) in arterial blood in normoxia (PaO_2_ 90 mmHg), while in hypoxia (PaO_2_ 45 mmHg), the increased Hb–O_2_ affinity increases arterial SO_2_ by 4–5% (5) (see also Table 3: PO_2_ required to obtain 90% saturation as a function of P_50_). A review on the influence of high Hb-O_2_-affinity on humans in hypoxia was recently published by Webb et al. (94). In COVID-19 patients, Valle et al. showed an association of an increased Hb-O_2_-affinity with a lower mortality rate (56). To define a therapeutic target, more research is needed with regards to balancing the benefits of more rapid pulmonary oxygen uptake (and increased arterial oxygenation) with the associated reduced rate of peripheral oxygen unloading. In this context, it will also be important to consider that the *in vivo* P_50_ alterations in the lungs will be dissimilar to the changes in peripheral tissue due to differing acid-base and temperature conditions (54). As such, in hypoxic respiratory failure an increased Hb-O_2_-affinity appears beneficial. However, the optimum degree of the desired ODC left-shift still needs to be identified.

### 9.4. By which means is it possible to achieve this target without detrimental side effects?

#### 9.4.1. “Old” blood transfusions

Storage of red blood cells for transfusion increases Hb-O_2_ affinity with time due to 2,3-BPG depletion (95, 96). Three large randomized controlled trials (RCTs) compared transfusion of fresh versus stored red blood cells in critically ill adults: the ABL (97), INFORM (98), and TRANSFUSE (99) trial with the latter recruiting almost 5,000 patients. None of these showed a difference in outcomes between the groups and in a meta-analysis no effect on mortality was found (100). However, the TRANSFUSE trial showed a lower mortality for older transfusions in a pre-specified subgroup with more severely ill patients. In ARDS patients, an observational study found no association between transfusion of older units and survival. Nevertheless, transfusion of “old” red cell units was associated with a lower chance for successful weaning from renal replacement therapy (101). Free hemoglobin acts as a potent vasoconstrictor, even at low concentrations (102). Indeed, transfusion of “old” red cell units was associated with increased plasma hemoglobin levels and increased pulmonary artery pressure (103). Furthermore, only a fraction of transfused red cells remains in the circulation for more than 24 h (104) and this proportion is lower following transfusion of “older” blood (105). In addition, storage of red cells alters their rheological properties unfavorably (106) and may disrupt physiologic vasodilatory responses (107). Thus, while it is possible to increase Hb-O_2_ affinity with the transfusion of 2,3-BPG depleted blood, multiple confounding properties and side effects of transfusions exist.

#### 9.4.2. 5-Hydroxymethylfurfural (5-HMF)

5-Hydroxymethylfurfural (5-HMF) is an aromatic aldehyde formed during non-enzymatic browning and caramelization of carbohydrate-containing foods after thermal treatment (the so called “Maillard” reaction) (108). Yalcin et al. showed that 5-HMF increases Hb-O_2_ affinity (93). Woyke et al. demonstrated this dose dependent effect *in vitro* in whole blood and discussed its potential use in COVID-19 patients (109, 110). Mahon et al. demonstrated that 5-HMF increased the Hb-O_2_ affinity in a swine model of hypoxia, resulting even in beneficial effects on pulmonary artery pressure and a trend toward improved mortality (111). Similar results were obtained by Lucas et al. (108) in a hamster model. In combination with α-ketoglutaric acid, 5-HMF has been shown to increase maximal aerobic capacity during the peri-operative period in lung surgery (112), and resulted in a predictable left-shift in healthy volunteers (109). Hence, while further research is still needed, 5-HMF (with or without α-ketoglutaric acid) is available and may represent a potential agent of choice to increase Hb-O_2_ affinity in patients with severe respiratory failure.

#### 9.4.3. Voxelotor (GBT440)

Voxelotor (previously known as GBT440), a specific agent for use in patients with sickle cell disease, has been approved by the US Food and Drug Administration (FDA) in 2019. Data from animal models had shown that GBT440 binds specifically to Hb, increases the Hb-O_2_ affinity, prevents sickling and prolongs red blood cell half-life (113). Voxelotor causes a dose dependent left shift in Hb-O_2_-affinity. Howard et al. (114) and the multicenter, randomized controlled HOPE trial (115) showed a disease-modifying potential. However, the lack of reduction of clinically important pain episodes has been criticized (77). Voxelotor has also been tested in healthy volunteers exercising under hypoxic conditions and showed the expected increase in oxygen content (116), however, it has not been trialed in respiratory failure or ARDS models.

#### 9.4.4. GBT1118

GBT1118, a voxelotor analogue, is a small molecule that reversibly binds to the NH_2_-terminal chain of Hb and increases Hb-O_2_-affinity. In a mouse model of ARDS, Putz et al. (117) showed that a single dose of GBT1118 improved oxygen saturation, severity of illness, and survival. the effect was associated with reduced hypoxia in the kidney and liver, but was independent of airspace inflammation and alveolar-capillary barrier permeability (117). Similar findings were reported by Dufu et al. (118). Thus, GBT1118 represents a promising agent to induce a left shifted ODC in respiratory failure once further research is available.

#### 9.4.5. Nebulized epoprostenol

Woyke et al. exposed venous blood samples from healthy volunteers to the pulmonary vasodilators epoprostenol and iloprost (119). With epoprostenol, they detected an increased Hb-O_2_ affinity in all subgroups under laboratory conditions, however, further research is required to validate this effect *in vivo*.

#### 9.4.6. Volatile anesthetic agents

According to Ronzani et al. (120), the volatile anesthetic agents isoflurane, desflurane and sevoflurane affect the ODC in a concentration dependent manner. While low to medium concentrations of isoflurane or desflurane were associated with a right shift, high concentrations of desflurane and sevoflurane increased Hb-O_2_ affinity. Sevoflurane might be of particular interest as it has been studied in ARDS patients where it not only improved oxygenation but also decreased levels of markers of epithelial injury and inflammation (121).

### 9.5. Conclusion concerning the treatment of COVID-19

While it is possible to increase Hb-O_2_ affinity with the transfusion of 2,3-BPG depleted blood, multiple confounding properties and side effects of transfusions exist. Meanwhile, several agents that specifically increase Hb-O_2_ affinity are available. The voxelotor analogue GBT1118 has shown reliable effects on the ODC, even with promising outcomes in murine respiratory failure models. Further research is still required; yet, 5-HMF (with or without α-ketoglutaric acid) is available and may represent a potential agent of choice to increase the Hb-O_2_ affinity in patients with severe respiratory failure. Finally, nebulized epoprostenol and sevoflurane (inhaled at high doses)–two agents which are already used in ARDS patients–increase Hb-O_2_ affinity under laboratory conditions, and studying whether these can be exploited in a therapeutic way may be of particular interest in the near future.

### 9.6. Treatment of COVID-19 in sickle cell disease

According to Safo and Kato (122) the main therapeutic strategy for sickle cell anemia should aim to stabilize the R-state of hemoglobin, which has higher oxygen affinity and would be expected to have slower kinetics of polymerization. 5-HMF forms a high-affinity Schiff-base adduct with HbS and inhibits red cell sickling by allosterically shifting oxygen equilibrium curves toward the left (123). Therefore, similar drugs as in the treatment of COVID-19 have been suggested, for instance 5-HMF and Voxeletor (124).

Another possible strategy seems to be the reduction of iron supply, because this element is important for replication of the virus (66). Interestingly the severety of COVID-19 in SCD children with sickle cell disease is low (80). In this paper the authors state that “COVID-19 presentation was mild in children and moderate in many SCD adults.”

The postulated therapeutic strategy by Safo and Kato (122) has stimulated the laboratory investigation of aromatic aldehydes, aspirin derivatives, thiols and isothiocyanates that can stabilize the R-state of hemoglobin *in vitro*. One representative aromatic aldehyde agent, 5-hydoxymethyl-2-furfural (5-HMF, also known as Aes-103) increases oxygen affinity of sickle hemoglobin and reduces hypoxia-induced sickling *in vitro* and protects sickle cell mice from effects of hypoxia. 5-HMF has completed pre-clinical testing and has entered clinical trials. The development of Hb allosteric modifiers as direct anti-sickling agents is an attractive investigational goal for the treatment of SCD.

## 10. General conclusion

SARS-CoV-19 damages red cells as early as during their production in the medulla ossea. Studies on the effects of COVID-19 on hemoglobin oxygen affinity have been performed in some hundred patients with contrasting results, probably due to varying methods and non-uniform groups of COVID-19 patients as well as comparison subjects. Most investigators have calculated half saturation pressures from single blood samples, only few have analyzed complete dissociation curves but with rather artificial methods. In the majority of severely ill patients a left shift of the curve was found, especially in those with a good prognosis. This change favors oxygen loading into the blood in the lungs. The cause for the changes is unknown because an analysis of red cell constituents was rarely performed.

In SCD the negative effects of COVID-19 seem to be mitigated, possibly because the effects of a SARS-CoV-2 infection counteract the unfavorable large right shift of the ODC by the hereditary illness. In the long term, further investigations will prove increasingly difficult, as most controls will no longer be real non-infected controls once a majority of the population has been infected by SARS-CoV-2 over the past years (125). Also, comparison with other illnesses may be misleading, if those also affect hemoglobin oxygen affinity.

## Author contributions

DB, DV, and WB wrote sections of the manuscript. WK contributed to each section. All authors contributed to conception and design of the study, manuscript revision, read, and approved the submitted version.
